# Presumed occult globe rupture resulting in sympathetic ophthalmia

**DOI:** 10.1007/s12348-011-0056-4

**Published:** 2011-12-27

**Authors:** Martin Galea, Kevin Falzon, Vikas Chadha, Graeme Williams

**Affiliations:** 1 Tennent Institute of Ophthalmology, Gartnavel General Hospital, 1053, Great Western Road, Glasgow, UK; 2 St. James University Hospital, Beckett Street, Leeds, UK

**Keywords:** Sympathetic ophthalmia, Occult, Globe rupture, Sub-conjunctival pigmentation

## Abstract

**Introduction:**

Sympathetic ophthalmia (SO) is an uncommon bilateral granulomatous panuveitis following uveal trauma to one eye. We present an unusual case of SO which resulted from presumed occult globe rupture following blunt trauma; and highlight the association of trauma and acquired external ocular pigmentation as a possible predictor for SO.

**Case report:**

Five weeks following blunt trauma to the left eye (OS), a 30-year-old patient presented complaining of spontaneous blurred vision (4/60) in the right eye (OD). In the OD, there was anterior chamber and vitreous inflammation (3+), multiple areas of serous retinal detachments, Dalen Fuchs spots, and optic disk swelling. In the OS, there was iridodialysis, post-traumatic acquired external ocular pigmentation suggestive of occult globe rupture.

This was diagnosed as SO and treated with systemic steroids and a steroid sparing agent; which resulted in resolution of the inflammation with improvement in the visual acuity.

**Conclusion:**

Sympathetic ophthalmia has been reported to occur following penetrating eye injury secondary to trauma and surgery, and also secondary to non-penetrating eye trauma. This case reports SO occurring after presumed occult globe rupture; and reinforces the association between acquired external ocular pigmentation and SO in the context of trauma.

## Introduction

Sympathetic ophthalmia (SO) is an uncommon bilateral granulomatous panuveitis following uveal trauma to one eye. The British Ophthalmology Surveillance Unit (BOSU) reported an incidence of SO of 0.03 per 100,000 [1].

We present an unusual case of SO which resulted from a presumed occult globe rupture following blunt trauma; and highlight the fact that acquired external ocular pigmentation in association with trauma can be a possible predictor for SO.

## Case report

**Fig. 1:**
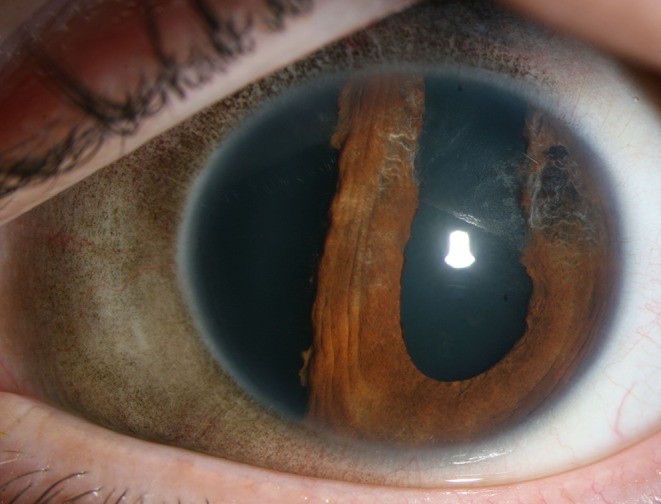
Iridodialysis and sub-conjunctival pigmentation in the left eye. (Photograph taken 5 weeks after the patient’s initial presentation)

A 30-year-old gentleman attended the eye casualty service complaining of blurred vision in the left eye (OS) following alleged blunt trauma during an assault 5 days previously. On presentation, the vision in the affected eye was 6/18. There was a superior sub-conjunctival hemorrhage, extensive iridodialysis (7 to 11 clock hours) (Fig. 1), anterior chamber inflammation (cells 2+) and hyphema. The intraocular pressure (IOP) was recorded at 14 and 15 mmHg in the right and left eye, respectively. The view of the left fundus was hazy, however, with a flat retina. Treatment was conservative with topical steroids.

**Fig. 2:**
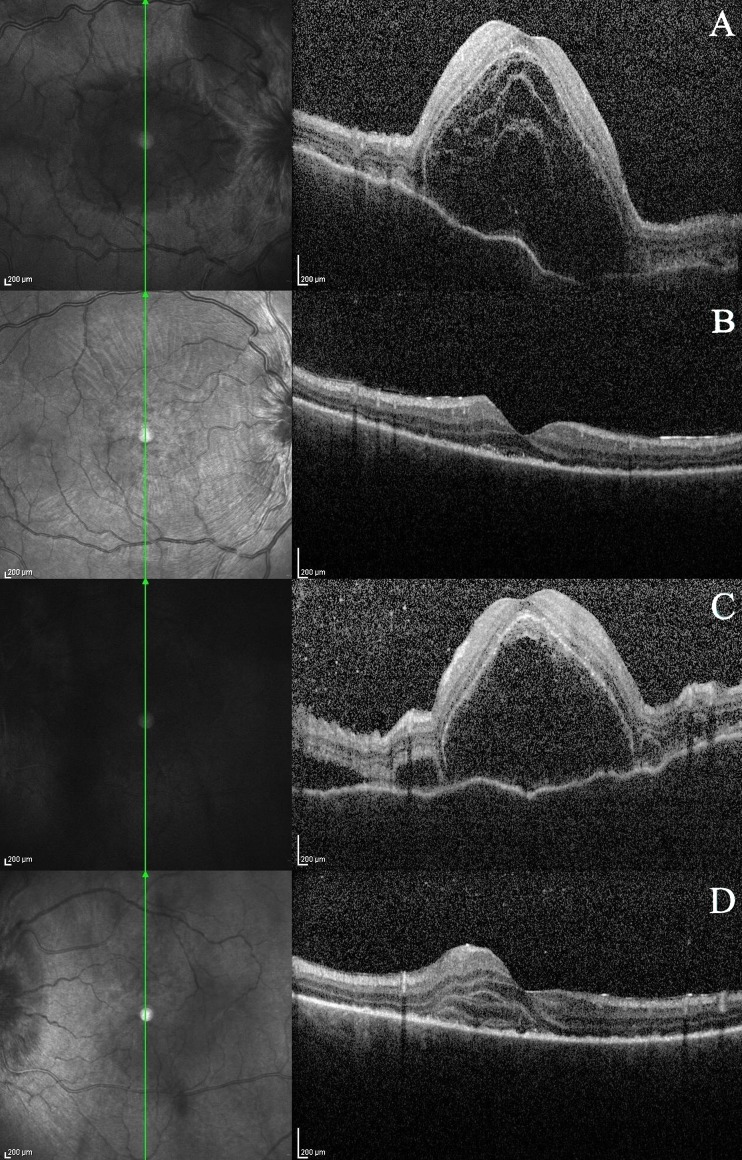
OCT images of **a** serous RD of the right macula pre-intravenous steroids; **b** significant improvement of the serous RD in the OD following intravenous steroids; **c** serous RD of the left macula pre-intravenous steroids; **d** significant improvement of the serous RD in the OS following intravenous steroids

**Fig. 3:**
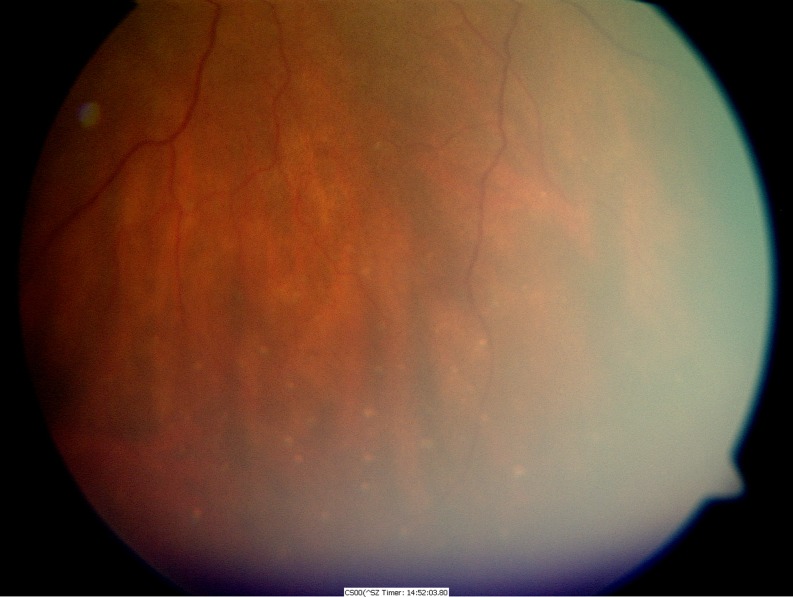
Dalen Fuchs nodules in the right eye

The patient failed to attend his follow-up reviews in the clinic. Five weeks later, the patient presented again to the eye casualty service complaining of blurred vision, pain, and photophobia in his right eye (OD). On this occasion, there was no history of trauma. The vision in the right eye was recorded at 4/60. There were inflammatory cells (cells 3+) in the anterior chamber associated with vitreous cells (cells 3+), multiple areas of serous retinal detachments (RD; Fig. 2a), discrete deep yellow lesions typical of Dalen Fuchs nodules (Fig. 3) and optic disk swelling. Although comfortable, there were inflammatory cells (cells 1+) in the anterior chamber of the left eye associated with vitreous cells (cells 1+). There was sub-conjunctival pigmentation superiorly and nasally (Fig. 1). Fundoscopy revealed multiple areas of serous RDs (Fig. 2c) and optic disk swelling in the same eye. The vision in the left eye was recorded at 2/60.

A diagnosis of sympathetic ophthalmia following presumed occult globe rupture was made. Other possible causes were considered including sarcoidosis, syphilis, and Vogt Koyanagi Harada syndrome (VKH). The patient did not give a history of respiratory problems. Serum calcium and ACE levels were within normal limits. Treponema serology was negative. With regards to VKH, the patient was a Caucasian and did not manifest any features of neurological deficit, alopecia, or poliosis. Furthermore, the occurrence of the ocular signs and symptoms in such close proximity to the history of trauma favored a diagnosis of SO.

The patient was admitted to hospital for pulsed intravenous methylprednisolone 1 g daily for 3 days. The patient’s vision improved to 6/6 bilaterally with the steroids. The inflammation subsided and the serous RDs improved (Fig. 2b, d). He was eventually discharged from the eye ward on 60 mg oral prednisolone and tacrolimus 1 mg BD. One month following discharge, the patient was still doing well with stable visual acuities.

However, during the subsequent visit (4 weeks later), it was noted that compliance with treatment was sub-optimal due to the social circumstances of the patient. This was reflected in diminishing visual acuities in both eyes (OD 6/24; OS 2/60) and reactivation of the bilateral granulomatous panuveitis. Attempts to re-admit the patient to hospital were declined. The patient has failed to attend all further follow-up reviews. This therefore limited the length of follow-up period to just 2 months post discharge from hospital.

## Discussion

Our case describes SO in a context suggestive of a self-sealing and occult globe rupture. Predictors of scleral rupture including a significantly reduced visual acuity, low intraocular pressure, and relative afferent pupillary defect were absent during the patient’s initial presentation [2].

The sub-conjunctival pigmentation superiorly and nasally in the left eye was consistent with acquired external ocular pigmentation. Such findings have been reported in the literature in association with globe rupture [3, 4].

Moreover, significant sub-conjunctival pigmentation has also been reported to be associated with SO in relation to pars plana vitrectomy [5]. The histopathological findings are those of deposition of melanin granules both extracellularly and within macrophages [5].

The combination of trauma, sub-conjunctival pigmentation, and onset of SO reinforces our suspicion of a presumed self-sealing and occult globe rupture. We postulate that hypotony was never present at the initial presentation and follow-up reviews because the presumed rupture was self-sealing and hence precluding a low IOP.

## Conclusion

The temporal association between the injury sustained to the left eye and resultant bilateral intraocular inflammation would suggest trauma as the precipitant factor. We therefore suggest that SO is the pathogenic process in this patient.

SO has been reported to occur following penetrating eye injury, non-penetrating eye trauma [6], and following intra-ocular surgery especially vitreoretinal [5].

This case demonstrates that it is likely that SO can occur following occult globe rupture as well; and highlights the fact that acquired external ocular pigmentation in association with trauma can be a possible predictor for SO.
